# Assessing Novel Drugs and Radiation Technology in the Chemoradiation of Oropharyngeal Cancer

**DOI:** 10.3390/medicines5030065

**Published:** 2018-06-27

**Authors:** Agostino Cristaudo, Mitchell Hickman, Charles Fong, Paul Sanghera, Andrew Hartley

**Affiliations:** 1 Department of Radiation Oncology, University of Pisa, 56100 Pisa PI, Italy; agostino.cristaudo@uhb.nhs.uk; 2 Hall-Edwards Radiotherapy Research Group, Queen Elizabeth Hospital, Birmingham B15 2TH, UK; mitchell.hickman@uhb.nhs.uk (M.H.); charles.fong@uhb.nhs.uk (C.F.); paul.sanghera@uhb.nhs.uk (P.S.)

**Keywords:** clinical target volume (CTV), mucositis, organ at risk (OAR), intensity modulated proton therapy (IMPT), PET/CT

## Abstract

Integrating immunotherapy, proton therapy and biological dose escalation into the definitive chemoradiation of oropharyngeal cancer poses several challenges. Reliable and reproducible data must be obtained in a timely fashion. However, despite recent international radiotherapy contouring guidelines, controversy persists as to the applicability of such guidelines to all cases. Similarly, a lack of consensus exists concerning both the definition of the organ at risk for oral mucositis and the most appropriate endpoint to measure for this critical toxicity. Finally, the correlation between early markers of efficacy such as complete response on PET CT following treatment and subsequent survival needs elucidation for biological subsets of oropharyngeal cancer.

## 1. Introduction

The identification of prognostic subgroups of patients with oropharyngeal cancer on the basis of a p16 expression and smoking status has led to attempts to de-escalate treatment in patients with a good prognosis and escalate treatment for patients with poor or intermediate prognosis tumours [1,2,3,4].

Controversy persists as to both the net quality of life benefit and the oncological safety of current de-escalation strategies [5]. Phase 2 trials examining the role of radiotherapy dose de-escalation after either neoadjuvant chemotherapy or surgery have shown the potential for such approaches, but the use of multiple treatment modalities may diminish the anticipated improvement in morbidity [6]. Large phase 3 trials where radiotherapy dose has not been altered but patients are randomised between synchronous cetuximab and cisplatin have completed recruitment but have not yet reported results [6]. Trials exploiting the reduced low dose bath associated with the use of intensity modulated proton therapy (IMPT) are underway or are being set up [7].

For patients with a poor or intermediate prognosis, phase 3 trials are ongoing to examine the effect of neoadjuvant or adjuvant immunotherapy and radiotherapy dose escalation [8]. 

The purpose of this non-systematic review is to examine four key areas of controversy in phase 2 and phase 3 oropharyngeal trials, namely the clinical target volume definition, oral cavity organ at risk (OAR) definition, measurement of acute mucosal reactions and early assessment of response with PET CT. These may both affect the reliability and reproducibility of results and be relevant to the timely production of data, particularly when considering the addition of novel systemic agents.

## 2. Clinical Target Volume Definition

The ultimate goal in a radiation plan is to include every malignant cell and exclude healthy uninvolved tissue. The clinical target volume (CTV) aids this concept by permitting an expansion beyond the known gross tumour volume (GTV) to include microscopic disease and particularly in the head and neck, to allow for uncertainties in defining the GTV. Note, the use of this CTV in the head and neck is different to the elective dose that aims to solely eradicate microscopic disease. Inter-clinician heterogeneity in outlining CTVs persists despite routine intensity modulated radiotherapy (IMRT) use. In response, an international consensus guideline was published in November 2017 concerning the contouring of CTVs for head and neck cancer [9]. The main principle of the guidelines is that the GTV is surrounded by a 5 mm rim of the high dose CTV, which in turn is surrounded by a further 5 mm rim of low or intermediate dose CTV. Various structures are then to be excluded from the low or intermediate CTV. Our interpretation of the text is given in Table 1, Table 2, Table 3 and Table 4. The radiation data on which the guidelines are based is not presented in detail, but the principles have been adopted by a number of international trial groups. Many current trials use a margin of 10 mm around the GTV for the high dose CTV. It will be important to consider this volumetric difference when comparing the toxicity and efficacy of future combinations using the international consensus guidelines with current and historical studies [8,10,11]. To a greater degree, toxicity improvements might be expected when results are compared with some early IMRT trials where much larger volumes were contoured for the high dose CTV to encompass whole anatomical subsites [12]. The evolution of contouring and techniques is discussed further below in the mucosal organ at risk section.

Given the international consensus it may seem reasonable to employ the new guidelines with the caveats regarding toxicity comparison and the necessity for an accurately definable GTV using MRI fusion. This may not be possible in patients in centres without MRI simulation or where there is uncertainty regarding the clinical/radiological extent of the GTV. 

Further considerations are necessary when attempting to compare IMRT with proton therapy, including intensity modulated proton therapy (IMPT). Interest in the use of IMPT for oropharyngeal cancer is increasing, buoyed by potential dosimetric advantages and early cohort experiences from single institutions. These suggest comparable efficacy and an improved toxicity profile compared to IMRT [13,14,15]. A phase III non-inferiority study comparing IMPT vs IMRT in stage III/IV oropharyngeal carcinoma is currently ongoing in the USA [7]. 

The issue of standardising CTV delineation is as relevant with IMPT, notwithstanding the unique treatment planning aspects for IMPT target volumes which cannot be based on geometric margin expansion alone, owing to increased range uncertainty with resultant increased sensitivity to set-up and anatomical changes. Degradation of the dose distribution for both the target volumes and OARs can be proportionately worse for IMPT compared to IMRT over the entire course of treatment, resulting in less than anticipated therapeutic benefits for IMPT [16,17]. Studies evaluating robust planning parameters specific to oropharyngeal cancer have recently emerged and robustness settings do influence the dose to OARs with possible clinical impact [18,19]. As the concept of a purely margin-based planning target volume (PTV) may not be appropriate for IMPT and uncertainties remain with regards to RBE, it is difficult to infer direct benefits from a modelling approach alone and randomised clinical evaluation will help to determine the actual level of benefit. However, to ensure the reproducibility of results, it is important to carefully define OARs and correlate dosimetric data with clinical outcomes for each technique. 

## 3. Oral Cavity OAR Definition

Although regarded as a severe and potentially dose limiting acute side effect of radiotherapy for oropharyngeal cancer, there is uncertainty in defining the delineation of the oral cavity as an OAR to enable oral mucositis reduction [20,21,22]. The reasons for this uncertainty can be elucidated from a consideration of the history of head and neck radiotherapy over the last 30 years.

In the era of 2D radiotherapy and early 3D conformal radiotherapy, potential for sparing of the oral cavity was limited. Models derived from trials conducted in this era show clear relationships between prescription dose converted to biologically effective dose (BED) or an equivalent dose in 2 Gray (Gy) fractions (EQD_2_) and the rate of grade 3 mucositis [23,24]. Attempts have also been made to model the additional effect on oral mucositis of synchronous chemotherapy [25,26]. 

The contralateral parotid gland geographically isolated from the high dose volume in a node negative neck and intuitively definable as an organ at risk, was the most obvious target to examine for clinical benefit with the advent of IMRT [12]. More recently research has centred on developing models to predict the swallowing function following radiotherapy [27,28,29]. The dependence of late swallowing function on various intuitive swallowing OARs, including the pharyngeal constrictor muscles and the potential for modest reductions in the incidence of late swallowing problems by reducing the dose to these structures, has been examined by modelling studies and is being examined in an ongoing randomised trial [10,30].

Only more recently, with the lower dose bath possible with proton therapy, has there been renewed interest in defining a relationship between dose to a putative oral mucosal OAR and acute mucositis. The difficulties in defining an appropriate endpoint for acute mucositis will be discussed below but the various OARs for the oral mucosa which have been proposed will now be considered. Much of this work has been carried out by investigators at the Royal Marsden Hospital who developed dose response curves for the incidence of grade 3 oral mucositis based on 144 patients from prospective trials [31]. The oral cavity OAR used was originally described by Eisbruch et al. with the intention of reducing xerostomia by reducing minor salivary gland dose with IMRT [32].

Due to concerns that the oral cavity contours proposed by Eisbruch et al. were oversimplified, the Royal Marsden group proposed the mucosal surface contour (MSC) [33,34]. This consisted of a 3 mm contour of all the mucosal surfaces in the oral cavity. The same group then successfully published NTCP models for grade 3 mucositis based on the original patients in the Bhide et al. study with an additional 207 patients from other prospective studies [22]. However, they found that neither the use of the MSC nor the use of machine learning or atlas-based methods improved the performance of the NTCP model [35,36]. Despite median dose to the oral mucosa being significant on multivariate analysis for predicting oral mucositis in their earlier atlas-based method, the Marsden Group concluded that high and intermediate dose to the original Eisbruch OAR should be reduced. RTOG trial protocols had previously recommended attempts to constrain the median dose to this structure. The use of the Common Toxicity Criteria Adverse Events (CTCAE) version 2 and 3 systems throughout the Marsden Group studies and their classification into the presence on at least 1 occasion of severe mucositis (grade 3 or 4) may explain the apparent serial radiobiological behaviour of the oral mucosa. Such a binary endpoint not taking in to account either the spatial distribution of severe mucositis or its duration is likely to be highly dependent on high dose to an oral cavity contour or indeed prescription dose to the high dose planning target volume. The MSC is not recommended for investigators trying to establish NTCP models except where the spatial distribution of mucositis is scored using an outcome measure such as the Oral Mucositis Assessment Scale [37]. 

International consensus guidelines for organs at risk have been published where for the “sake of simplicity” an “extended oral cavity” was defined partly on the basis of the xerostomia structure proposed by Hoebers and Eisbruch et al. [38,39]. In these guidelines, oral mucosa lateral to the mandible was defined as a Buccal Mucosa OAR and anterior to the mandible as part of a Lips OAR. The surface of both these structures had previously been included in the MSC. 

The original Marsden NTCP model for mucositis has recently been tested on a proton treated patient cohort [40]. The model did not perform as well as anticipated. The authors, who did not include any of the Marsden group, concluded that this may be due to the subjective nature of the physician scoring of acute mucositis and the lack of data on spatial distribution of mucositis due to the use of CTCAE versions 2 and 3. Another possibility for the poor performance is that the OAR for mucositis is called “buccal mucosa” in the model validation study for protons and it is uncertain how this correlates with the original Eisbruch OAR or the international consensus “buccal mucosa” OAR.

When considering NTCP models for mucositis or other endpoints, it is important to consider not only the OAR which has been employed but the patient population used to generate the model. Both the Bhide cohort and the proton validation cohort employed patients from several anatomical sites including but not exclusively oropharynx cases, which is the focus of this current review [31,40]. In addition, both populations were subject to reactive rather than prophylactic feeding tube placement. However, Sanguineti et al. reported on the effect of dose to an oral mucosa OAR on feeding tube dependence longer than 3.3 months [27]. In a reactive feeding tube group, who were patients treated with radiotherapy alone, there was a steep dose response curve for the volume of oral mucosa receiving >9.5 Gy per week and the risk of feeding tube dependency at >3.3 months. Conversely, in a prophylactic feeding tube group where all patients received synchronous chemotherapy, the dose response curve was shallow and the relationship not statistically significant (*p* = 0.055). Consistent with these findings is the lack of a relationship between acute mucositis and dysphagia at 6 months from the Dutch NTCP model group with a historic prophylactic feeding tube policy [41]. This may also explain the exclusion of dose to an oral cavity OAR as a significant factor in the Dutch NTCP 6 month dysphagia model developed to assess benefit from both dysphagia sparing IMRT and IMPT [29]. The relationship with high doses to the oral mucosa in the first Sanguineti cohort is consistent with the observations from the Marsden Group regarding the importance of a high and intermediate dose rather than a median dose and support a serial model of behaviour of the oral mucosal OAR when physician scoring of worst mucosal appearance is considered.

Few studies have considered the oral cavity OAR exclusively in oropharyngeal patients. Yahya et al. were unable to find a relationship between the duration of grade 3 mucositis scored using CTCAE version 3 and dose to four different oral mucosal OARS [42]. The 65 patients in the study were treated with synchronous chemotherapy in a centre with a reactive feeding tube policy to the same prescription dose. The lack of a relationship in this study may be explained by the shallow dose response curve seen by Sanguineti in the presence of synchronous chemotherapy. In addition, such a homogenous population may require more patients to produce a robust model than models derived from multiple anatomical sites. 

In the future for the sake of consistency, investigators should employ the international consensus guideline Extended Oral Cavity OAR even if previous NTCP models may not directly relate to this [38]. The importance of developing future NTCP models using a consistent oral cavity OAR, a relevant population and a sensitive and meaningful mucosal endpoint will be further emphasised in the following section.

## 4. Measurement of the Acute Mucosal Reaction

The acute mucosal reaction during and following radiotherapy has been scored in three main ways. Historically, grading systems such as the CTCAE version 3 or RTOG, scoring the reaction on the basis of physician assessment of mucosal appearance have been employed. In such systems confluent mucositis greater than 1–1.5 cm in diameter have been scored as grade 3. Such systems have the advantage of being simple to use. In addition, early radiobiological models linking mucositis and prescription dose were based on such physician scored objective systems [23,24]. More complex systems for scoring mucositis such as the Oral Mucositis Assessment Scale (OMAS) where the extent of mucositis is incorporated by assigning a grade to different anatomical regions within the mouth have subsequently been developed [37].

More recently, systems relying on physician scoring of patient symptoms have been developed including the WHO system where grade 3 equates with ulceration sufficient to prevent the patient swallowing food and the CTCAE version 4 where grade 3 is defined by the patient requiring strong analgesia for their mucositis [21,43]. 

Finally, there are many questionnaires for patients to report their own assessment of symptoms and quality of life during and after treatment [44]. The National Cancer Institute have produced a patient reported outcome measure (PRO-CTCAE) where patients simply grade the severity of their mucositis over the last week as mild, moderate, severe and very severe [45]. 

Given the evolution of the scoring systems described above, it is important to realise that most of the radiobiological data discussed in the previous section is derived from physician scored mucosal appearance grading systems. Hickman et al. compared a mucosal appearance grading system, CTCAE version 3, with a physician scoring of patient symptoms system, CTCAE version 4 [43]. Out of 555 episodes of mucosal scoring using both systems, different grades were recorded 228 (41%) times i.e., CTCAE version 3 grading is significantly different than CTCAE version 4. 

In addition, as discussed above, early linear quadratic models relating mucositis to prescription dose expressed as BED and later NTCP models considered the binary endpoint of the presence or absence of grade 3 mucositis at any time point during or in the weeks following radiotherapy. Denham et al. found an association between the duration of acute grade 3 mucositis and the incidence of late mucosal toxicity in a study comparing conventional with accelerated radiotherapy [46]. In view of this finding, it is important that studies continue to report the duration of acute grade 3 mucositis by mucosal appearance. 

Chung et al. re-analysed a randomised trial examining the effect of 5% phenylbutyrate mouthwash on the rates of physician scored (RTOG and OMAS) and patient reported mucositis during head and neck chemoradiation [21]. The initial study showed no benefit on the mucosal endpoints used but the subsequent analysis showed that despite mucosal appearance and patient reported symptoms being similar between the arms of the study, there were less radiotherapy interruptions and a greater compliance to synchronous chemotherapy in the experimental arm. Therefore, they demonstrated that both types of systems could be confounded by such mucositis related events and that treatment compliance should from an integral part of mucositis assessment. 

Two randomised trials have examined the effect of palifermin, a keratinocyte growth factor on mucositis [47,48]. In the study of Henke et al. in postoperative patients receiving chemoradiation the primary endpoint was the incidence of severe mucositis (grade 3–4) as scored by mucosal appearance using the RTOG scale. The secondary endpoints importantly included duration of severe mucositis, patient reported mouth or throat soreness ((MTS) which corresponds to the CTCAE mucosal PRO above) and mucositis related events including radiotherapy interruption and chemotherapy compliance. Incidence (51% v 67%; *p* = 0.027) and median duration of physician scored severe mucositis (4.5 v. 22 days; *p* = 0.037) were significantly reduced in the experimental arm but there was no significant difference in patient reported MTS or radiation interruptions. The results of the second study by Le et al. were very similar demonstrating a lack of concordance between physician and patient scored endpoints.

Gussgard et al. investigated the relationship between the mucositis extent using the OMAS system and patient reported outcomes. In this small study, they found that there was a clearer relationship between the severity of mucositis at any location (i.e., worst mucositis grade) than with the extent of mucositis [49]. This is again suggestive of a serial behaviour of the oral mucosa when using both physician scored and patient reported outcomes.

Hickman et al. have recently presented data not only repeating their observation of the discrepancies between CTCAE version 3 and 4 but also between these systems and the PRO-CTCAE mucosal grading. Although there were a high number of discrepancies in individual scoring episodes the median duration of grade3 mucositis on version 3 and version 4 and at patient reported level of “severe” was similar at 7.5–8 weeks. In addition, they found a relationship between both the volume of high dose CTV and chemotherapy corrected BED and the duration of patient reported mucositis level “severe” suggesting that the latter may be a useful endpoint in escalation studies [50].

The most common form of de-escalation, namely replacing cisplatin with cetuximab, has been examined in three randomised studies which are yet to report [6]. These trials were based on the observation in the registration study that cetuximab did not increase the duration of physician scored severe mucositis when compared with radiotherapy alone [20]. However, patient reported mucositis has not been presented.

In summary, given the discrepancies in grading seen between physician and patient reporting systems, it is recommended that both simple physician and patient reported mucosal scoring be used in studies examining new interventions. In addition, careful documentation of mucositis related events, for example, compliance with radiotherapy and chemotherapy and total dose of opiate used should be reported. For studies examining the benefit of particles or a reduced clinical target volume, a scoring system measuring mucositis extent may also be included given the apparent serial behaviour of the oral mucosa when scored by worst physician scored appearance or patient reported outcome [51].

## 5. The Role of PET CT in Response Assessment Following Treatment

In addition to assessing the toxicity of any new intervention in the definitive chemoradiation of oropharyngeal cancer, a reliable and early mechanism for detecting changes in efficacy is desirable. ^18^FDG PET CT scanning has been used extensively to assess response following radical chemoradiation in head and neck cancer [52]. In the PET NECKstudy, PET CT guided follow up of the irradiated neck was associated with a similar survival and lower costs when compared with planned neck dissection [53]. Many studies of the role of PET CT in this setting focus on the high negative predictive value for subsequent primary site and nodal recurrence across a spectrum of head and neck anatomical sites [54]. This underlines the utility of PET CT as a modality for comparing efficacy after novel interventions. 

Eleven studies, including seven retrospective and four prospective studies, evaluating the role of PET in assessment of response to chemoradiation for oropharyngeal squamous cell carcinoma were identified and are summarised in Table 5 [55,56,57,58,59,60,61,62,63,64,65]. In accordance with the known prognostic significance of p16 status seven of the eleven studies differentiate between p16 positive and negative oropharyngeal tumours [1]. This is essential for reference with future escalation and de-escalation studies. Three studies focused exclusively on p16 positive patients [56,64,65].

The timing of the first PET evaluation after completion of chemoradiotherapy may affect the response rate and this is still a matter of debate [66,67,68]. Scans performed before 3 months may result in a higher incidence of false positive results which may lead to unnecessary psychological morbidity. In the majority of the eleven studies summarised in Table 2, PET CT was performed at 3 months. Prestwich et al. performed PET CT 4 months after the end of CRT, while in two studies PET CT was performed at only 6 and 9 weeks post treatment [57,60,63]. A randomised comparison of PET CT performed at the current standard of 3 months versus a later date might answer the key question as to whether a later assessment might reduce false positives without compromising surgical salvage.

Mak et al. report response rates for both p16 positive and p16 negative tumours. In addition, response rates are provided for both the primary site and in the neck [61]. A higher response rate for p16 positive cancers (90% at the primary and 93% in the neck) was reported compared to p16 negative (78% and 83%).This manner of reporting by p16 status (and if possible by smoking status), broken down by neck and primary site should be the paradigm for future studies, as it provides the most useful comparative data for future investigators. It is disappointing that to date, so few studies have documented responses in this way. In addition, the use of standardised criteria (Hopkins criteria) in differentiating likely complete response (Hopkins score 1–3) from likely residual disease (Hopkins score 4–5) is required for adequate comparison of response rates [69].

## 6. Conclusions

In future trials in oropharyngeal cancer the international consensus guidelines on target volume and organ at risk delineation should be employed despite the uncertainties described in this paper. Careful consideration should be given to choosing outcome measurements, but a combination of physician scored, patient reported and mucositis related adverse events systems should be used. For, ^18^FDG PET CT response data to be meaningful it should be reported by biological subset and cite the response at the primary site and in nodal disease using the Hopkins criteria.

## Figures and Tables

**Table 1:** Summary of structures which may be included (✔) or where possible excluded (x) from intermediate and low dose CTV—an interpretation of the text of the international consensus guidelines for tonsil tumours.

Site	Stage	Superior Constrictor	Para-Pharyngeal SPACE	Base of Tongue/Mobile Tongue	Medial Pterygoid	Lateral Pterygoid	Mandible	Retromolar Trigone	Hard Palate
Tonsil	T1	✔	x	x	x	x	x	x	x
	T2	✔	✔	✔	x	x	x	x	x
	T3	✔	✔	✔	✔	x	x	x	x
	T4	✔	✔	✔	✔	✔	✔	✔	✔

**Table 2:** Summary of structures which may be included (✔) or where possible excluded (x) from intermediate and low dose CTV- an interpretation of the text of the international consensus guidelines for base of tongue tumours.

Site	Stage	Pre-Epiglottic Space	Hyo-Glossus	Para-Pharyngeal SPACE	Adjacent Nodes e.g., Levels Ib/II; Retropharyngeal	Lateral portion Superior Pharyngeal Constrictor	Mobile Tongue	Medial portion Superior Pharyngeal Constrictor	Hyoid	Larynx/Hypopharynx
Base of Tongue	T1	x	x	x	x	x	x	x	x	x
	T2	✔	✔	✔	✔	✔	✔	x	x	x
	T3	✔	✔	✔	✔	✔	✔	x	x	x
	T4	✔	✔	✔	✔	✔	✔	✔	✔	✔

**Table 3:** Summary of structures which may be included (✔) or where possible excluded (x) from intermediate and low dose CTV- an interpretation of the text of the international consensus guidelines for soft palate tumours.

Site	Stage	Hard Palate	Lateral Pharyngeal Wall	Para-Pharyngeal Space	Medial Pterygoid	Mobile Tongue	Nasopharynx	Nasal Cavity
Soft palate	T1		x	x	x	x	x	x
	T2	✔	✔	✔	x	x	x	x
	T3	✔	✔	✔	✔	x	x	x
	T4	✔	✔	✔	✔	✔	✔	✔

**Table 4:** Summary of structures which may be included (✔) or where possible excluded (x) from intermediate and low dose CTV- an interpretation of the text of the international consensus guidelines for posterior pharyngeal wall tumours.

Site	Stage	Pharyngeal Constrictor	Longus Muscles	Prevertebral Fascia	Vertebral Body
Posterior pharyngeal wall	T1–T3	✔	x	x	x
	T4	✔	✔	✔	✔

**Table 5:** Rates of complete response on FDG PET scan following chemoradiation for oropharyngeal cancer from published series (gaps in table represent data not presented) (RT: radiotherapy, N: number of patients; CR: complete response; CR T: complete response primary tumour, CR N: complete response nodes).

				Whole Study				p16+ve				p16-ve			
Reference	Study	Timing of FDG PET (Weeks)	RT Dose	N	CR	CR T	CR N	N	CR	CR T	CR N	N	CR	CR T	CR N
[65]	Prospective	12	60 Gy/30#	79			44 (56%)	79 (100%)			44 (56%)				
[58]	Retrospective	15 (median)	70 Gy/35#	50	39 (78%)										
[55]	Retrospective	12.4 (median)	65 Gy/30#	146	90 (62%)			96 (66%)				35 (24%)			
[59]	Prospective	13.5 (median)	70 Gy/35#	50			45 (90%)	32 (64%)				7 (14%)			
[62]	Prospective	12.4 (median)	70 Gy/35#	36		36 (100%)	28 (76%)								
[64]	Retrospective	13.4 (median)	70 Gy/35#	98				98 (100%)		87 (88%)	91 (93%)				
[60]	Retrospective	9		61	48 (79%)			50 (82%)	40 (80%)			11 (18%)	8 (73%)		
[61]	Retrospective	12 (mean)	70 Gy/35#	48		41 (85%)	43 (89%)	30 (63%)		27 (90%)	28 (93%)	18 (37%)		14 (78%)	15 (83%)
[56]	Retrospective	12 (median)	70 Gy/35#	67		67 (100%)	59 (88%)	67 (100%)		67 (100%)	59 (88%)				
[63]	Retrospective	16.8 (median)	70 Gy/35#	30		25 (83%)	26 (87%)								
[57]	Prospective	6	60 Gy/30	18	14 (78%)										
